# Supplementary material for: Sex differences in absolute and relative changes in muscle size following resistance training in healthy adults: a systematic review with Bayesian meta-analysis
Source: PeerJ. 2025 Feb 25;13:e19042. doi: 10.7717/peerj.19042 (PMC11869894; doi:10.7717/peerj.19042)
Supplement: Supplemental Information 2 [file peerj-13-19042-s002.docx]

**Describe the audience it is intended for:**

We intend to inform the wider health and fitness community about the similar potential in muscle hypertrophy from resistance training between males and females. The article is also intended for researchers to plan future studies that assess aspects of our article that were hypothesis generating.

**Describe how disagreements were resolved, and identify the referee:**

The article identification process was completed independently (to reduce any bias during this process) by two authors (MR and JF) with any disagreement resolved through discussion with LH, who acted as a referee to make the final decision. Finally, the authors (MR and JF) reviewed the full text to determine eligibility for inclusion based on the inclusion criteria.

* The above text as now been added to the article.
